# Preventive Dental Care and Oral Health of Children and Adolescents With and Without Heart Conditions — United States, 2016–2019

**DOI:** 10.15585/mmwr.mm7106a1

**Published:** 2022-02-11

**Authors:** Karrie F. Downing, Lorena Espinoza, Matthew E. Oster, Sherry L. Farr

**Affiliations:** ^1^Division of Birth Defects and Infant Disorders, National Center on Birth Defects and Developmental Disabilities, CDC; ^2^Division of Oral Health, National Center for Chronic Disease Prevention and Health Promotion, CDC; ^3^Division of Pediatric Cardiology, Department of Pediatrics, Children’s Healthcare of Atlanta, Emory University, Atlanta, Georgia.

Approximately 900,000 U.S. children have heart conditions, such as congenital heart disease (*1*). These children might be at increased risk for life-threatening infective endocarditis from oral bacteria in the bloodstream (*2*). Therefore, preventive dental care (i.e., check-ups, dental cleaning, radiographs, fluoride treatment, or sealant) to maintain oral health is important. Oral health status and receipt of preventive dental care were compared between children with heart conditions (2,928) and without (116,826) using population-based 2016–2019 National Survey of Children’s Health (NSCH) data. Approximately 83% of children with and 80% without heart conditions received preventive dental care in the past year (p = 0.06). Children with heart conditions were more likely than were those without to have poor oral health (17.2% versus 13.7%; p = 0.02) and teeth in fair or poor condition (9.9% versus 5.3%; p<0.01). Among those with a heart condition, having low household income; an intellectual or developmental disability; and no well-child visit or medical home were associated with poor oral health. Receipt of preventive dental care was higher among children aged ≥6 years and those with insurance. Public health practitioners and health care providers can implement strategies (e.g., parent and patient education and collaboration between pediatricians, dentists, and cardiologists) to improve oral health and care among children with heart conditions, especially those with fewer resources and intellectual or developmental disabilities.

The NSCH is an annual parent-reported survey to evaluate health, well-being, and related factors among U.S. persons aged 0–17 years.* One child from each household was randomly selected to be the subject of the survey. The overall weighted response rates per year for the 2016–2019 surveys were 40.7%, 37.4%, 43.1%, and 42.4%, respectively. Parents were asked whether a health care provider ever said their child had a heart condition. Only parents of children and adolescents aged 1–17 years with any teeth were asked about their child’s oral health^†^ and receipt of dental care^§^. Children missing any data of interest were excluded. Characteristics of excluded and included children and adolescents were compared using Wald chi-square tests. Crude and adjusted associations between heart condition status and oral health and preventive dental care were assessed using Wald chi-square tests and with the predicted marginals obtained from logistic regression models. Among children and adolescents with a heart condition, adjusted prevalence ratios (aPRs) evaluated associations between their characteristics and receipt of preventive dental care, fair or poor condition of teeth, and one or more indicators of poor oral health. All models included sex, age, and race/ethnicity. Certain models also included household income as a percentage of federal poverty level and having intellectual or developmental disabilities, including Down syndrome.

Analyses were conducted using SAS-callable SUDAAN (version 11; RTI International). Design parameters accounting for complex sampling, weight, and nonresponse bias produced nationally representative, population-based estimates. Among 126,996 children and adolescents in NSCH aged ≥1 year who had one or more teeth, 6.3% were excluded because information on heart condition (0.3%), oral or dental outcomes (2.9%), or other characteristics (3.1%) was missing; although there was no difference in heart condition status between included and excluded persons, excluded persons more commonly had poor oral health and less commonly had preventive dental visits. In total, 2,928 children and adolescents with heart conditions (representing 1.4 million U.S. children and adolescents) and 116,826 children and adolescents without heart conditions (representing 6.4 million U.S. children and adolescents) were included.

Children and adolescents with heart conditions were less likely to be Hispanic or uninsured and more likely to be non-Hispanic White and have public insurance, intellectual or developmental disabilities, special health care needs, and well-child visits (Table 1). Approximately 84% of those with a heart condition received any dental care in the past 12 months (K Downing, CDC, unpublished data, 2022), and 83% received preventive dental care (Figure). Among children and adolescents who received preventive dental care, the majority received dental check-ups and dental cleanings (95% each), whereas application of sealant was least common (25%). Children and adolescents with a heart condition were more likely than were those without to receive preventive dental care overall as well as each of the individual services, although some CIs overlapped. Children and adolescents with a heart condition were approximately twice as likely to have teeth in fair or poor condition (10%) as were those without a heart condition (5%). Seventeen percent of children and adolescents with a heart condition had one or more indicators of poor oral health during the past 12 months. Decayed teeth or cavities (14%) was the most prevalent indicator of poor oral health. Prevalence for all indicators was higher among children and adolescents with a heart condition than among those without, although some CIs overlapped.

**TABLE 1:** Characteristics of persons aged 1–17 years with and without a heart condition — National Survey of Children’s Health,* United States, 2016–2019

Characteristic	Parental report of a heart condition			
	Never (n = 116,826)		Ever (n = 2,928)	
	No.	Weighted % (95% CI)	No.	Weighted % (95% CI)
**Sex**				
Male	60,398	51.1 (50.4–51.7)	1,607	52.5 (48.8–56.3)
Female	56,428	48.9 (48.3–49.6)	1,321	47.5 (43.7–51.2)
**Age group, yrs**				
1–5	30,304	28.6 (28.0–29.2)	715	26.7 (23.6–29.9)
6–11	36,866	35.6 (35.0–36.3)	914	37.7 (33.9–41.6)
12–17	49,656	35.8 (35.2–36.4)	1,299	35.7 (32.3–39.1)
**Race/Ethnicity**				
Black, non-Hispanic	7,024	13.1 (12.6–13.6)	175	12.7 (10.3–15.6)
White, non-Hispanic	81,661	51.3 (50.6–51.9)	2,141	60.1 (56.2–63.9)
Hispanic	13,284	24.9 (24.2–25.6)	279	17.8 (14.5–21.6)
Multiracial/Other^†^	14,857	10.7 (10.4–11.0)	333	9.5 (7.6–11.7)
**Insurance coverage**				
Any private	89,487	63.3 (62.6–64.0)	2,119	59.7 (55.6–63.6)
Public only	22,520	30.3 (29.6–31.0)	717	36.3 (32.3–40.4)
None	4,819	6.4 (6.0–6.8)	92	4.1 (2.8–5.8)
**Federal poverty level^§^**				
<100%	12,483	19.6 (19.0–20.2)	370	23.4 (19.6–27.8)
100%–199%	18,527	21.7 (21.1–22.3)	493	19.3 (16.5–22.3)
200%–399%	36,185	27.6 (27.1–28.2)	959	29.1 (26.1–32.3)
≥400%	49,631	31.1 (30.6–31.6)	1,106	28.3 (25.5–31.2)
**Has an intellectual or developmental disability**	7,776	6.5 (6.2–6.8)	697	24.2 (21.4–27.3)
**Has special health care needs^¶^**	27,000	19.2 (18.7–19.7)	1,533	47.9 (44.2–51.6)
Attended well-child visit**	96,817	79.6 (79.0–80.1)	2,639	89.6 (87.1–91.6)
**Has a medical home^††^**	64,104	48.6 (47.9–49.2)	1,555	48.7 (44.9–52.4)

* The National Survey of Children’s Health is weighted to be representative of the U.S. population of noninstitutionalized persons aged ≤17 years. https://www2.census.gov/programs-surveys/nsch/technical-documentation/methodology/NSCH-Guide-to-Multi-Year-Estimates.pdf

^†^ “Other” category includes respondents who self-identified as Asian, American Indian or Alaska Native, Native Hawaiian or other Pacific Islander, or mixed race.

^§^ Based on U.S. Department of Health and Human Services poverty guidelines.

^¶^ Special health care needs are defined as one or more of the following five conditions: needing prescription medicine; having more health care encounters than other children their age; having limitations compared with other children their age; needing physical, occupational, or speech therapy; or having an emotional, developmental, or behavioral problem in need of counseling or treatment. To be classified as special health care needs, these conditions must be related to a medical, behavioral, emotional, developmental, or other health condition that lasts or is expected to last ≥12 months.

** Attended a well-child visit in the past 12 months.

^††^ https://www.childhealthdata.org/docs/medical-home/mhmanual_withappendices-updated-12-7-10-pdf

**FIGURE:**
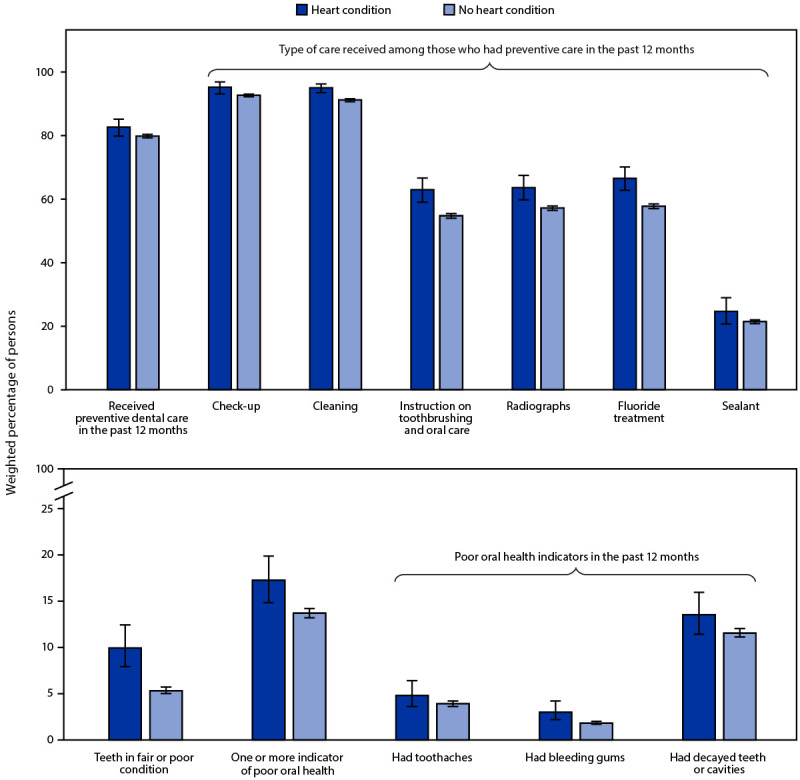
Weighted prevalence* of preventive dental care and oral health indicators^†^ of persons aged 1–17 years with and without a heart condition — National Survey of Children’s Health, United States, 2016–2019 * The National Survey of Children’s Health is weighted to be representative of the U.S. population of noninstitutionalized persons aged ≤17 years. https://www2.census.gov/programs-surveys/nsch/technical-documentation/methodology/NSCH-Guide-to-Multi-Year-Estimates.pdf † Parent reported that child had frequent or chronic difficulty with toothaches, bleeding gums, decayed teeth, or cavities in the past 12 months.

The adjusted prevalence of receipt of preventive dental care was similar in children and adolescents with and without a heart condition (Table 2). Among those with a heart condition, receipt of preventive dental care was more prevalent among older age groups than youngest (6–11 years: aPR = 1.3; 12–17 years: aPR = 1.3). Point prevalence of preventive dental care was lowest among those without health insurance (55.7%; aPR = 0.6). Prevalence was also lower among those without a medical home^¶^ (aPR = 0.9).

**TABLE 2:** Associations between oral health and dental care and ever having a heart condition among persons aged 1–17 years with and without heart conditions and associations with sociodemographic and health characteristics among persons with a heart condition — National Survey of Children’s Health,* United States, 2016–2019

Characteristic	Received preventive dental care in the past 12 months		Teeth in fair or poor condition		One or more indicators of poor oral health^†^	
	Weighted % (95% CI)	aPR^§^ (95% CI)	Weighted % (95% CI)	aPR^§^ (95% CI)	Weighted % (95% CI)	aPR^§^ (95% CI)
**Among all persons (N = 119,754)**						
**Heart condition^¶^**						
Ever	82.6 (79.8–85.1)	1.0 (1.0–1.1)	9.9 (7.9–12.4)	1.8 (1.4–2.3)	17.2 (14.8–19.8)	1.2 (1.0–1.4)
Never	79.8 (79.2–80.3)	Ref	5.3 (5.0–5.7)	Ref	13.7 (13.2–14.2)	Ref
**Among persons with a heart condition (n = 2,928)**						
Sex^¶,^**						
Male	80.3 (76.1–83.9)	Ref	11.8 (8.8–15.7)	Ref	17.0 (14.0–20.5)	Ref
Female	85.1 (81.3–88.2)	1.0 (1.0–1.1)	7.9 (5.6–11.1)	0.9 (0.6–1.3)	17.3 (13.8–21.5)	1.1 (0.8–1.5)
Age group, yrs^¶,^**						
1–5	66.4 (60.1–72.2)	Ref	9.7 (5.9–15.7)	Ref	14.7 (10.4–20.3)	Ref
6–11	90.8 (86.4–93.9)	1.3 (1.2–1.5)	9.5 (6.5–13.7)	1.2 (0.7–2.0)	18.2 (14.3–22.9)	1.3 (0.9–1.9)
12–17	86.0 (81.3–89.6)	1.3 (1.1–1.4)	10.6 (7.6–14.6)	1.3 (0.8–2.1)	17.9 (14.3–22.2)	1.2 (0.8–1.9)
Race/Ethnicity^¶,^**						
Black, non-Hispanic	77.9 (68.6–85.0)	1.0 (0.9–1.1)	16.5 (9.1–28.0)	1.1 (0.6–1.9)	24.3 (16.1–34.9)	1.5 (0.9–2.3)
White, non-Hispanic	83.4 (80.2–86.3)	Ref	8.5 (6.5–11.1)	Ref	13.5 (11.3–16.2)	Ref
Hispanic	88.5 (81.6–93.1)	1.0 (1.0–1.1)	6.8 (3.6–12.2)	0.8 (0.4–1.5)	22.2 (15.1–31.2)	1.5 (1.0–2.3)
Multiracial/Other^††^	72.3 (59.3–82.3)	0.9 (0.8–1.0)	16.3 (8.4–29.2)	1.7 (0.9–3.1)	21.1 (14.5–29.8)	1.5 (1.0–2.2)
Insurance coverage**						
Any private	85.5 (82.6–88.0)	Ref	7.8 (6.0–10.2)	Ref	15.9 (13.1–19.2)	Ref
Public only	80.8 (75.1–85.4)	1.0 (0.9–1.0)	11.7 (8.0–16.9)	1.0 (0.7–1.5)	19.4 (15.1–24.5)	1.0 (0.7–1.4)
None	55.7 (36.8–73.1)	0.6 (0.5–0.9)	25.1 (10.0–50.4)	2.5 (1.1–5.4)	15.7 (7.5–30.0)	0.8 (0.4–1.7)
Federal poverty level^¶,^**^,§§^						
<100%	78.6 (70.3–85.1)	0.9 (0.8–1.0)	20.0 (13.2–29.1)	2.9 (1.7–4.9)	23.1 (16.8–31.0)	1.7 (1.1–2.6)
100%–199%	81.0 (74.7–86.0)	0.9 (0.9–1.0)	7.4 (4.9–11.0)	1.2 (0.7–2.1)	18.5 (13.3–25.2)	1.4 (0.9–2.2)
200%–399%	83.9 (78.9–87.8)	1.0 (0.9–1.1)	7.9 (5.5–11.4)	1.4 (0.9–2.4)	17.1 (13.3–21.7)	1.4 (1.0–2.1)
≥400%	85.7 (81.1–89.3)	Ref	5.5 (3.6–8.2)	Ref	11.4 (8.4–15.3)	Ref
Has an intellectual or developmental disability^¶,^**						
Yes	78.3 (71.8–83.7)	1.0 (0.9–1.1)	25.8 (19.9–32.8)	4.7 (3.0–7.4)	25.2 (20.0–31.2)	1.7 (1.3–2.3)
No	84.0 (80.9–86.6)	Ref	4.9 (3.3–7.0)	Ref	14.6 (12.1–17.5)	Ref
Has special health care needs^¶,^**^,¶¶^						
Yes	81.8 (77.4–85.6)	1.0 (0.9–1.1)	15.1 (11.5–19.5)	1.2 (0.7–2.1)	20.7 (17.3–24.7)	1.2 (0.8–1.6)
No	83.3 (79.6–86.4)	Ref	5.2 (3.5–7.7)	Ref	13.9 (10.9–17.6)	Ref
Attended well-child visit^¶,^**^,^***						
Yes	83.5 (80.6–86.0)	Ref	8.9 (7.1–11.1)	Ref	16.9 (14.5–19.7)	Ref
No	75.0 (63.2–84.0)	0.9 (0.8–1.0)	19.1 (9.7–34.2)	1.9 (1.2–3.2)	19.4 (12.3–29.1)	1.1 (0.7–1.7)
Has a medical home^¶,^**^,^ ^†††^						
Yes	87.9 (85.1–90.3)	Ref	5.6 (4.0–7.9)	Ref	16.2 (13.1–20.0)	Ref
No	77.5 (72.9–81.6)	0.9 (0.8–0.9)	14 (10.6–18.4)	1.9 (1.3–2.9)	18.0 (14.7–21.9)	1.0 (0.7–1.3)

**Abbreviations:** aPR = adjusted prevalence ratio; Ref = referent group.

* The National Survey of Children’s Health is weighted to be representative of the U.S. population of noninstitutionalized persons aged ≤17 years. https://www2.census.gov/programs-surveys/nsch/technical-documentation/methodology/NSCH-Guide-to-Multi-Year-Estimates.pdf

^†^ Parent reported that child had frequent or chronic difficulty with toothaches, bleeding gums, decayed teeth, or cavities in the past 12 months.

^§^ Multivariable model includes sex, age, and race/ethnicity.

^¶^ Model also includes percentage of the federal poverty level.

** Model also includes having intellectual or developmental disabilities.

^††^ “Other” category includes respondents who self-identified as Asian, American Indian or Alaska Native, Native Hawaiian or other Pacific Islander, or mixed race.

^§§^ Based on U.S. Department of Health and Human Services poverty guidelines.

^¶¶^ Defined as having one or more of the following five conditions: needing prescription medicine, having more health care encounters than other children their age; having limitations compared with other children their age; needing physical, occupational, or speech therapy or having an emotional, developmental, or behavioral problem in need of counseling or treatment. To be classified as special health care needs, these conditions must be related to a medical, behavioral, emotional, developmental, or other health condition that lasts or is expected to last ≥12 months.

*** Attended a well-child visit in the past 12 months.

^†††^ https://www.childhealthdata.org/docs/medical-home/mhmanual_withappendices-updated-12-7-10-pdf

Children and adolescents with a heart condition were more likely to have teeth in fair or poor condition (aPR = 1.8) and to have one or more poor oral health indicators (aPR = 1.2) than were those without a heart condition (Table 2). For both children and adolescents with teeth in fair or poor condition (aPR = 1.4) and those with one or more poor oral health indicators (aPR = 1.1), results were attenuated, but the former remained elevated, after adjusting for presence of intellectual disabilities (K Downing, CDC, unpublished data, 2022). Among children and adolescents with a heart condition, the prevalence of having teeth in fair or poor condition was highest among those with intellectual or developmental disabilities (25.8%; aPR = 4.7), those without insurance (25.1%; aPR = 2.5), and those living at <100% of the federal poverty level (20.0%; aPR = 2.9). This prevalence was elevated among persons without well-child visits (aPR = 1.9) and without a medical home (aPR = 1.9). The percentage of children and adolescents with one or more poor oral health indicators was highest among those with an intellectual or developmental disability (25.2%; aPR = 1.7) and was elevated among those living at <100% of the federal poverty level (aPR = 1.7).

## Discussion

In this large, population-based sample from the 2016–2019 NSCH, approximately 10% of children and adolescents with a heart condition had teeth in fair or poor condition, and 17% had one or more indicators of poor oral health, such as toothaches, bleeding gums, or cavities in the past 12 months. Furthermore, one in six had not received preventive dental care in the past 12 months. Prevalence of preventive dental care was consistently higher among children with a heart condition than among children without, although some differences did not reach statistical significance. Prevalence of poor oral health was also higher among children with a heart condition, although some differences were not statistically significant.

Some small, non-U.S., clinic-based studies have reported that children with congenital heart defects have worse oral health than children without heart defects (*3*–*6*), whereas others suggest no difference (*7*,*8*). Factors associated with preventive dental care and oral health among children with a heart condition have been less studied. In a 2016 NSCH analysis among all U.S. children, preventive dental care was similarly associated with older age and having insurance (*9*). Better condition of teeth was associated with well-child visits, although not with household income. In other literature, children with intellectual or developmental disabilities (who account for approximately one in five children with heart conditions in NSCH) had some of the highest rates of poor oral health (*10*).

The findings in this report are subject to at least five limitations. First, all data were parent-reported and not clinically confirmed. Second, sample size limited the ability to examine outcomes by heart condition severity, and data on heart condition type (congenital or acquired) were not collected. Third, some children might have had heart conditions in the past that were resolved. Fourth, 6% of surveys were excluded for missing data but are not expected to affect findings. Finally, only data from 2019 and earlier were available at the time of analysis, and receipt of dental treatment and oral health might have changed since then.

Children and adolescents with a heart condition, particularly those with intellectual disabilities, were more likely than those without a heart condition to have teeth in fair or poor condition. Approximately one in six children with a heart condition had toothaches, bleeding gums, or decay, and approximately one in six had not received preventive dental care during the past 12 months; although rates of some outcomes were similar to those without a heart condition, poor oral health and missed preventive dental care might have additional health implications for children with heart conditions. Among children and adolescents with a heart condition, oral health was notably worse for those with intellectual or developmental disabilities, those living in poverty, and those without insurance. These findings could guide strategies, such as parent and patient education and collaboration between pediatricians, dentists, and cardiologists, to improve oral health and care among children with heart conditions, especially those with fewer resources and intellectual or developmental disabilities.

**Summary**

**What is already known about this topic?**

U.S. children with heart conditions might be at increased risk for infective endocarditis from oral bacteria; however, little is known about their oral health.

**What is added by this report?**

During 2016–2019, only 83% of persons aged 1–17 years with heart conditions received preventive dental care. However, 17% had symptoms of poor oral health during a 12-month period, and 10% had teeth in fair or poor condition. Those with lower household incomes and intellectual and developmental disabilities had worse oral health.

**What are the implications for public health practice?**

Public health practitioners and health care providers can implement strategies to improve oral health and care among children with heart conditions, especially those with fewer resources and intellectual or developmental disabilities.

## Footnotes

* https://mchb.hrsa.gov/data/national-surveys

† To assess oral health, parents were asked about the condition of their child’s teeth and experience with the following indicators of poor oral health during the past 12 months: frequent or chronic difficulty with toothaches, bleeding gums, or decayed teeth or cavities.

§ To assess receipt of preventive dental care, parents were first asked if the child saw a dentist or oral health care provider for any oral health care during the past 12 months. If yes, they were asked if the child saw the oral health care provider for preventive dental care. Among children who received preventive dental care, the following were examined: check-ups, cleaning, instruction on tooth brushing and oral health care, radiographs, fluoride treatment, or sealant.

¶
https://www.childhealthdata.org/docs/medical-home/mhmanual_withappendices-updated-12-7-10-pdf
